# CCL21-CCR7 promotes the lymph node metastasis of esophageal squamous cell carcinoma by up-regulating MUC1

**DOI:** 10.1186/s13046-015-0268-9

**Published:** 2015-12-15

**Authors:** Mo Shi, Dong Chen, Dong Yang, Xiang-yan Liu

**Affiliations:** Department of Thoracic Surgery of Shandong Provincial Hospital Affiliated to Shandong University, Jinan, 250021 China; Department of Oncology of Jinan Central Hospital Affiliated to Shandong University, Jinan, 250013 China

## Abstract

**Background:**

CCR7 and MUC1 are correlated with lymph node metastasis in ESCC, but the role of MUC1 in the CCR7-induced lymphatic metastasis and the underlying molecular mechanism is still unclear.

**Methods:**

The expression of CCR7 and MUC1 was detected in the ESCC samples by IHC, and the clinical significance of CCR7 and MUC1 in ESCC was analyzed. The expression of CCR7 and MUC1 in ESCC cell lines was detected by qRT-PCR and western blot. The effect of CCL21 on the migration and invasion of ESCC cells was determined by transwell assay. The activity of MUC1 promoter was determined by luciferase reporter assay. The activation of Erk, Akt and Sp1 was detected by western blot and the binding of Sp1 to the MUC1 promoter was determined by ChIP.

**Results:**

The co-expression of CCR7 and MUC1 was detected in 153 ESCC samples by IHC, and both were correlated with lymph node metastasis, regional lymphatic recurrence and poor prognosis. Correspondingly, increasing levels of MUC1 mRNA and protein were detected in the ESCC cell lines KYSE410 and Eca9706 after treatment with CCL21 in a time- and dose-dependent manner. Furthermore, silencing MUC1 could remarkably suppress the invasion and migration of ESCC cells induced by CCL21. Moreover, heterologous CCR7 promoted the invasion and migration of KYSE150 and up-regulated MUC1 expression. Increasing levels of activated ERK1/2 and Akt were detected in KYSE410 after treating the cells with CCL21, and inhibiting the activation of ERK1/2 but not Akt caused the increased transcription of MUC1. Finally, the phosphorylation of Sp1 induced by ERK1/2 and subsequent increases in the binding of Sp1 to the muc1 promoter at −99/−90 were confirmed to cause the up-regulation of MUC1 induced by CCL21-CCR7.

**Conclusions:**

Our findings suggested that MUC1 plays an important role in CCL21-CCR7-induced lymphatic metastasis and may serve as a therapeutic target in ESCC.

## Background

Esophageal squamous cell carcinoma (ESCC) is one of the most common digestive tumors worldwide [1]. Surgery still remains the first choice of treatment for resectable ESCC, but the therapeutic effect is not always favorable, and the 5-year survival rate of patients is approximately 30–50 %; more than half of patients develop recurrence within 2–3 years after undergoing surgery [2, 3]. TNM staging is the main parameter used to predict recurrence and prognosis, but it sometimes lacks sensitivity and accuracy. Therefore, finding relevant biological molecules may assist in identifying patients at high risk for recurrence, and elucidating the underlying molecular mechanism would present clinical advantages in the treatment of this disease.

The process of cancer metastasis consists of a series of sequential and interrelated steps, including detachment from primary sites, intravasation, survival in the circulation and translocation to target organs, extravasation and colonization [4, 5]. However, very little is known about the molecular mechanisms that regulate cancer cells’ directional invasion into specific organs. According to the homing theory, the metastasis of certain tumor cells to specific organs results from chemotaxis: the chemokines highly expressed in target organs could attract and capture tumor cells by binding with the chemokine receptors expressed on the surface of tumor cells [6, 7]. As a member of chemokine receptor family, C-C chemokine receptor type 7 (CCR7) is mainly located on the membrane of mature dendritic and T cells, and it could induce the “homing” of dendritic and T cells to the lymph node by binding with its specific ligands CCL19 and CCL21, which are highly expressed in the endothelium of lymphatic vessels and secondary lymph nodes [8, 9]. Interestingly, studies have identified the up-regulation of CCR7 in various types of malignant tumors, such as breast cancer, gastric cancer, and prostate cancer, and have revealed its function in promoting lymph node metastasis [10–12].

MUC1 is a transmembrane heterodimer protein with two subunits: the MUC1 N-terminal subunit (MUC1-N) contains variable numbers of tandem repeats that are extensively glycosylated. MUC1-N associates with the cell surface by binding to the transmembrane MUC1 C-terminal subunit (MUC1-C), which is mainly located on the apical borders of normal epithelial cells [13]. The aberrant expression of MUC1 has been well documented in various cancers and is correlated with advanced tumor progression and metastasis potential; furthermore, several fundamental works revealed that MUC1-C may function as an oncogene in the progression of cancers by interacting with many biological molecules, such as ICAM-1, β-catenin, EGFR, c-Src, and p65, to promote cell motility, adhesion, proliferation, and survival [14–17]. Therefore, the overexpression of MUC1 may predict a more aggressive biological behavior in several types of cancers.

Our previous studies revealed that the expression of CCR7/MUC1 is correlated with regional lymphatic recurrence in pN_0_ ESCC patients [18, 19]; however, there are few reports of MUC1 expression in ESCC and no available information on the cross-talk between MUC1 and CCR7 in the progression of lymphatic metastasis. In this study, we hypothesized that the CCL21-CCR7 axis may facilitate the lymphatic metastasis of ESCC via MUC1, further explored the role of MUC1 and CCR7 in lymphatic metastasis, and elucidated the underlying molecular mechanism.

## Methods

### Patients

A total of 153 ESCC patients who underwent esophagectomy in the Department of Thoracic Surgery at the provincial hospital affiliated with Shandong University from January 2006–June 2009 were enrolled in this study. The inclusion criteria were as follows: (1) Squamous cell carcinoma of the middle thoracic esophagus diagnosed by postoperative pathology with staging from I–III; (2) no preoperative chemotherapy or radiotherapy; (3) no contraindications to surgery, and all patients underwent Ivor-Lewis esophagectomy with complete resection: no residual tumor cells were present on upper or lower cutting edge as verified by pathology, lateral margins with no residual focus to the naked eye, and two-field lymph node dissection; (4) a complete 5-year follow-up, and the first location of recurrence was regional lymphatic metastasis; and (5) no severe preoperative complications. TNM staging was determined by the criteria established by the International Union Against Cancer (UICC) in 2009 and lymph node dissection was undertaken according to the lymph node mapping system for esophageal cancer established by the American Joint Committee on Cancer (AJCC) in 1997. Written informed consent was obtained from all patients, and the study protocol was approved by the Institutional Ethics Committee of the provincial hospital affiliated with Shandong University according to Guide for Chinese Ethics Review Committees.

### Cell lines and culture

The human ESCC cell lines Eca9706, KYSE150, KYSE410 and KYSE450 were from the Cancer Institute and Hospital, Chinese Academy of Medical Sciences, China. The cells were cultured in Roswell Park Memorial Institute (RPMI) 1640 enriched with 1 % penicillin/streptomycin (sigma, USA) and 10 % fetal bovine serum (HyClone, USA). Cell culture plates were maintained in humidified incubators at 37 °C in a 5 % CO_2_ incubator.

### Real-time RT-PCR

Total cell RNA was extracted using the RNAiso Plus (TaKaRa, Japan) according to the manufacturer’s instructions. The purity of RNA was measured and determined by UV spectrophotometry; the OD260/280 value was 1.8–2.0. Quantitative real-time RT-PCR was performed using a LightCycler 480 II Real-Time PCR System (Roche, Frankfurt, Germany). The cycling conditions were as follows: 5 min at 95 °C for preincubation and 45 amplification cycles of 10 s at 95 °C, 10 s at 60 °C and 10 s at 72 °C with the SYBR® Premix Ex Taq™ (TaKaRa, Japan) according to the manufacturer’s instructions. PCR primers were as follows: MUC1, 5′-TCA GCT TCT ACT CTG GTG CAC AA-3′ (forward) and 5′-ATT GAG AAT GGA GTG CTC TTG CT-3′ (reverse); GAPDH, 5′-AGA AGG CTG GGG CTC ATT TG-3′ (forward) and 5′-AGG GGC CAT CCA CAG TCT TC-3′ (reverse). PCR was repeated at least twice in all samples. The expression level of MUC1 mRNA was calculated using a ratio of MUC1 mRNA against GAPDH mRNA.

### Immunohistochemistry

The immunohistochemistry studies of MUC1 and CCR7 were performed by the streptavidin peroxidase method. The formalin-fixed, paraffin-embedded tissues were cut into 4-μm sections. The sections were deparaffinized and incubated with 3 % hydrogen peroxide. Specific mouse polyclonal anti-MUC1 antibody (Abcam, UK) and specific rabbit polyclonal anti-CCR7 antibody (Abcam, UK) were used at a dilution of 1:100 and incubated at 4 °C overnight. The subsequent steps were followed according to the instructions of the secondary biotinylated antibody kit (Zhongshan Biotech, China). As negative controls, the primary antibody was replaced by the phosphate buffered saline. All sections were examined by two independent pathologists who were blinded to the clinical data. The immunohistochemical score (IHS) was calculated by combining the proportion score (percentage of positively stained cells) with the staining intensity score. The proportion score ranged from 0 to 4, as follows: 0 (<5 %), 1 (5–24 %), 2 (25–49 %), 3 (50–74 %), and 4 (≥75 %). The staining intensity was scored as follows: 0 (negative), 1 (weak), 2 (moderate), and 3 (strong). The proportion score and staining intensities score were then multiplied to generate the IHS for each case. Cases with IHS ≥8 were considered to be positive for the expression of CCR7 or MUC1.

### Stable transfection by lentivirus

The lentiviral vector containing specific siRNA targeting MUC1 (si-MUC1, specific sequence targeting MUC1: 5′-ACC AAT TTC TCG GAC ACT T-3′)/Sp1 (si-Sp1, specific sequence targeting Sp1: 5′-TTT CGC GAA GTA CTC CTC ACT-3′), full-length cDNA of CCR7 (−CCR7) or a nonsense sequence serving as a scrambled negative control (si-NC/-NC) were obtained from Genechem (Shanghai, China). The ESCC cell lines were transfected by the lentivirus according to the manufacturer’s instructions with an MOI of 50:1. Twenty-four hours later, the medium containing lentivirus was replaced with complete medium containing puromycin (1 μg/ml) to remove the stable transfected cells.

### Immunoblotting

The total protein of cultured cells was extracted and measured as described previously. To detect the phosphorylated protein, the phosphatase inhibitor was used with a working concentration of 1 mM in the total protein extraction. The proteins were transferred to Hybond-P polyvinylidene difluoride membranes in trans-buffer containing 25 mM Tris and 185 mM glycine (pH 8.3) together with 20 % methanol. After transfer, the membranes were blocked for 1 h in blocking buffer (TBS containing 0.1 % Tween-20, TBST, supplemented with 5 % non-fat dry milk) and the membranes were incubated overnight at 4 °C with antibodies (MUC1,MUC1-C and p-Sp1 were obtained from Abcam, UK; p-ERK, ERK, p-akt and akt were obtained from CST, USA; GAPDH and β-actin were obtained from Santa Cruz, USA) in blocking buffer. After being washed three times with TBST, the membranes were incubated with secondary antibody conjugated with horseradish peroxidase (HRP) anti-mouse/rabbit IgG (Santa Cruz, USA) for 1 h at room temperature. The bands were visualized by an enhanced chemiluminescence (ECL) detection system (LAS-4000 MINI System, GE, California, USA).

### Cell immunofluorescence

The starved KYSE410 or Eca9706 cells were treated with PBS or CCL21 (Peprotech, USA) for 24 h, and the cells were fixed by 4 % paraformaldehyde for 20 min and incubated with Triton X-100 (0.3 %). After being blocked by goat serum for 30 min, the cells were incubated with the primary antibodies anti-MUC1-C or anti-Sp1 overnight. Then, the cells were washed with PBS three times and incubated with PE-conjugated secondary antibody (Santa Cruz, USA) for 1 h. After being washed with PBS three times and incubated with DAPI for 15 mint, coverslips were sealed with a drop of Prolong Gold antifade reagent (Invitrogen, USA). Images were acquired by laser scanning microscopy (Eclipse Ti, Nikon, Tokyo, Japan) and analyzed by NIS-Elements D 3.2.

### Cell migration and invasion assay

Cell migration and invasion experiments were assayed in triplicate using a 24-well transwell setup and polycarbonate nucleopore filters with an 8-μm pore size (Merck Millipore Bioscience, Germany). Prior to each experiment, cells were deprived of FBS for 24 h. For invasion assays, the inserts were pre-coated with Matrigel (BD Biosciences, Belgium) diluted 1:10 in serum-free Ham’s F10, and the Matrigel was allowed to solidify for 1 h at 37 °C. For migration assays, the inserts were left uncoated. Each upper well was loaded with starved 2 × 10^5^cells in a total volume of 200 μL of serum-free medium. The lower wells of the chamber were loaded with 600 μL of 1640 medium. Invasion assays were allowed to proceed for 36 h, whereas migration assays were incubated for 12 h. For migration assays, CCL21 (200 ng/mL) or PBS was added to the lower chamber, while for invasion assay, CCL21 (200 ng/mL) or PBS was added to the upper chamber. Any cells remaining on top of the insert were removed by scraping. Migrated cells attached to the underside were fixed for 10 min in methanol and stained with ethanol-based crystal violet solution. Cells were observed under a microscope (Eclipse Ti, Nikon, Tokyo, Japan) and all cells in five random visual fields in the middle of the membrane were counted.

### Transient transfection and luciferase assay

KYSE410 cells were seeded in RPMI 1640 with 10 % FBS in 24-well plates and incubated for 24 h at 37 °C, 5 % CO_2_, to 70–80 % confluence. Medium was replaced with serum-free RPMI 1640, and the cells were starved for another 24 h. The medium was replaced by RPMI 1640, 1 % FBS, and the cells were transfected using X-tremeGENE HP DNA Transfection Reagent (Roche, Germany) according to the manufacturer’s instructions. The MUC1 promoter-firefly luciferase reporter plasmid (MUC1-pGL2b), MUC1 promoter −97/−96(GG → AA) mutant-firefly luciferase reporter plasmid (MUC1 mutant-pGL2b), the empty pGL2b vector and phRL-TK internal control plasmid encoding Renilla luciferase and were constructed as described previously [16]. Briefly, sample DNA was mixed with 2.0 μL of X-tremeGENE and diluted with OPTI-MEMI (Invitrogen, USA) to a final volume of 100 μl. After15 min of incubation at room temperature, 600 μL of the transfection mixture was added to each well. After 6 h, the medium containing transfection mixture was replaced with RPMI 1640 with 1 % FBS. Then, the cells were treated with 200 ng/ml CCL21 or PBS as control for 12 h at 37 °C. To inhibit the activation of ERK1/2/Akt, the transfected KYSE410 cells were pretreated with U0126/MK2206 (Cell Signaling, USA) for 30 min before CCL21 treatment. Luciferase activity was determined using the Dual Luciferase assay system (Promega) according to the manufacturer’s instructions for the SpectraMax M2 (Molecular Devices, California, USA). Luciferase activity driven by the MUC1 promoter was normalized to the internal control by calculating the ratio of firefly luciferase activity to Renilla luciferase activity in each sample and was expressed as the percentage of MUC1 promoter activity relative to control samples.

### Chromatin immunoprecipation assay

ChIP assay was performed using the Pierce™ Agarose ChIP Kit according to the manufacturer’s instructions (Thermo Fisher, USA). Briefly, 2 × 10^6^ KYSE410 cells pretreated with PBS or CCL21 were treated with 1 % formaldehyde at room temperature for 10 min to cross-link DNA with its binding proteins. Then, the glycine solution was added and incubated at room temperature for 5 min to stop the cross-linkage reaction. After being washed with ice-cold PBS three times, the nuclei of the cross-linked cells were extracted and then sheared with 0.3 μL MNase (10U/μL) in a 37 °C water bath for 15 min. After centrifugation, the nuclei were resuspended in 50 μL nuclear extraction buffer and incubated in ice for 15 min. Then, 45 μL digested chromatin was added to 450 μL IP dilution buffer and incubated with Sp1 antibody (Abcam, UK) or rabbit IgG as a negative control at 4 °C overnight with the remaining 5 μL of digested chromatin as input. After incubation, the immunoprecipitated sample was mixed with 20 μL Protein A/G plus agarose resin and incubated for 1 h at 4 °C. After being washed three times, 150 μL IP elution buffer was added to the resin and incubated at 65 °C for 30 min, then mixed with 6 μL of 5 M NaCl and 2 μL of 20 mg/mL proteinase K to reverse the protein-DNA cross linkage; while the input sample was mixed with 150 μL of IP elution buffer, 6 μL of 5 M NaCl and 2 μL of 20 mg/mL proteinase K and incubated at room temperature. The eluted IP and input samples were placed at 65 °C for 1.5 h, and then the DNA was extracted and precipitated with ethanol for the PCR test. The MUC1 primer was as follows: 5′-TCT TAT TTC TCG GCC GCT CTG CTT-3′ (forward), 5′-TGG GTA GGG TAC AAG GGC TCT AAT-3′ (reverse). The automatic electrophoresis gelatin image formation analysis system (Alphalmager™ 2000, Alpha Innotech, California, USA) was used to determine the gray value of the amplified bands.

### Statistical analysis

SPSS 19.0 was used to create databases for data analysis. For continuous variables, Student’s *t*-test was performed. The correlation between CCR7/MUC1 protein expression and pathological parameters was determined by the chi-square test or Fisher’s exact probability test (two-tailed tests). Survival and recurrence rates were calculated by the Kaplan-Meier method and analyzed by the log-rank test.

## Results

### Co-expression of CCR7 and MUC1 in ESCC tissue

**Fig. 1:**
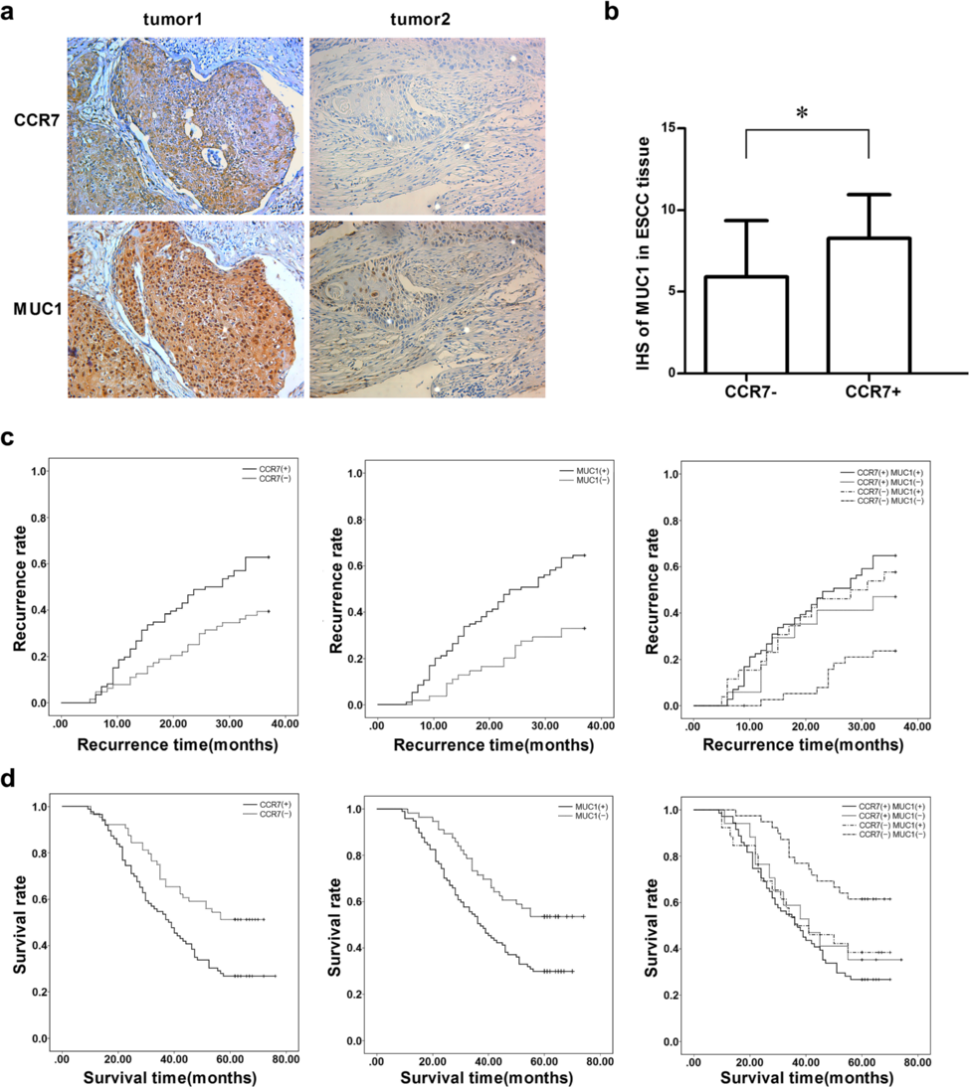
Co-expression of CCR7 and MUC1 in ESCC sample. **a** The expression of CCR7 and MUC1 in ESCC detected by IHC; **b** the immunoreactivity score of MUC1 in group with CCR7 positive expression and CCR7 negative expression group; **c** the 3-year regional recurrence curve of patients with CCR7 and MUC1 positive/negative expression; **d** the 5-year survival curve of patients with CCR7 and MUC1 positive/negative expression

To determine the correlation of CCR7 and MUC1 in ESCC tissue, we detected the expression of CCR7 and MUC1 by IHC in 153 ESCC samples. The immunostaining showed that CCR7 was mainly detected on the membrane and in the cytoplasm of tumor cells. MUC1 was mainly located in the nuclei and cytoplasm while partially on the membrane. The positive expression of CCR7 was detected in 88 samples with the remaining 65 samples staining negative, and the positive expression of MUC1 was detected in 97 samples with the remaining 56 samples staining negative. The IHS of MUC1 in group with positive CCR7 expression was 8.011 ± 2.673 (mean ± SD), while the IHS of MUC1 in group with negative CCR7 expression was 5.773 ± 3.454 (mean ± SD). The MUC1 expression was higher in ESCC samples with positive CCR7 expression than in the negative samples (*P* < 0.001, Fig. 1a, b).

### The expression of CCR7 and MUC1 correlated with the lymph node metastasis, regional lymphatic recurrence and poor prognosis

In 89 patients with lymph node metastasis, there were 60 (67.4 %) patients with positive CCR7 expression, and 67 (75.3 %) patients with positive MUC1 expression. In 64 patients without lymph node metastasis, there were 29 (45.3 %) patients with positive CCR7 expression and 22 (34.4 %) patients with positive MUC1 expression. The expressions of CCR7 and MUC1 were both positively correlated with lymph node metastasis (Table 1). Meanwhile, in the 89 patients with lymph node metastasis, 53 patients co-expressed CCR7 and MUC1 (59.6 %), seven patients only expressed CCR7 (13.2 %), 18 patients only expressed MUC1 (20.2 %), and 11 patients did not express CCR8 or MUC1 (12.4 %). The co-expression of CCR7 and MUC1 was also positively correlated with lymph node metastasis (Table 2).

**Table 1:** Clinical characteristics and its relationship with CCR7/ MUC1 expression

Clinical characteristic	CCR7 positive expression	*P value*	MUC1 positive expression	*P value*
Gender		0.45		0.85
Male	69/116		74/116	
Female	19/37		23/37	
Age(year)		0.86		0.2
≥50	63/108		72/108	
<50	25/45		25/45	
Length of tumor(cm)		0.98		0.08
<3 cm	20/35		20/35	
3 ~ 5 cm	42/72		50/72	
>5 cm	26/46		27/46	
Invasion depth		0.27		0.07
T1	11/25		11/25	
T2	44/76		49/76	
T3 + T4a	33/52		37/52	
Lymphatic metastasis		0.01		<0.001
Yes	60/89		67/89	
No	28/64		30/64	
Differentiation		0.14		0.42
Well/moderate	34/67		35/67	
Low	54/86		62/86	
Weight loss		0.5		0.16
Yes	15/23		18/23	
No	73/130		79/130	
Regional lymphatic recurrence		0		<0.001
Yes	54/78		61/78	
No	41/75		39/75

**Table 2:** Clinical characteristics and its relationship with CCR7 & MUC1 expression

Clinical characteristic	CCR7+ MUC1+	CCR7+ MUC1-	CCR7- MUC1+	CCR7- MUC1-	*P value*
Gender					0.46
Male	57/116	12/116	17/116	30/116	
Female	14/37	5/37	9/37	9/37	
Age(year)					0.61
≥50	52/108	11/108	20/108	25/108	
<50	19/45	6/45	6/45	14/45	
Length of tumor(cm)					0.65
<3 cm	16/35	4/35	4/35	11/35	
3 ~ 5 cm	34/72	8/72	16/72	14/72	
>5 cm	21/46	5/46	6/46	14/46	
Invasion depth					0.170
T1	9/25	2/25	2/25	12/25	
T2	35/76	9/76	14/76	18/76	
T3 + T4a	27/52	6/52	10/52	9/52	
Lymphatic metastasis					<0.001
Yes	53/89	7/89	18/89	11/89	
No	18/64	10/64	8/64	28/64	
Differentiation					0.06
Well/moderate	26/67	8/67	9/67	24/67	
Low	45/86	9/86	17/86	15/86	
Weight loss					0.2
Yes	12/23	3/23	6/23	2/23	
No	59/130	14/130	20/130	37/130	
Regional lymphatic recurrence					<0.001
Yes	46/78	8/78	15/78	9/78	
No	25/75	9/75	11/75	30/75	

The 3-year recurrence rates of patients with positive and negative CCR7 expression were 59.1 and 32.3 %, respectively (*P* < 0.01). The 3-year recurrence rates of patients with positive and negative MUC1expression were 59.8 and 26.8 % (*P* < 0.001), respectively. The 3-year recurrence rate of patients with positive CCR7 and MUC1 expression, positive CCR7 and negative MUC1 expression, negative CCR7 and positive MUC1 expression, and negative CCR7 and MUC1 expression were 73.2, 47.1, 57.7 and 38.5 %, respectively (*P* = 0.004, Fig. 1c).

The 5-year survival rates of patients with positive and negative CCR7 expression were 28.4 and 52.3 %, respectively (*P* < 0.01). The 5-year survival rates of patients with positive and negative MUC1 expression were 29.9 and 53.6 % (*P* < 0.01), respectively. The 5-year survival rates of patients with positive CCR7 and MUC1 expression, positive CCR7 and negative MUC1 expression, negative CCR7 and positive MUC1 expression, and negative CCR7 and MUC1 expression were 26.8, 35.3, 38.5 and 61.5 %, respectively (*P* < 0.001, Fig. 1d).

### CCL21-CCR7 up regulated MUC1 expression in ESCC cell lines

**Fig. 2:**
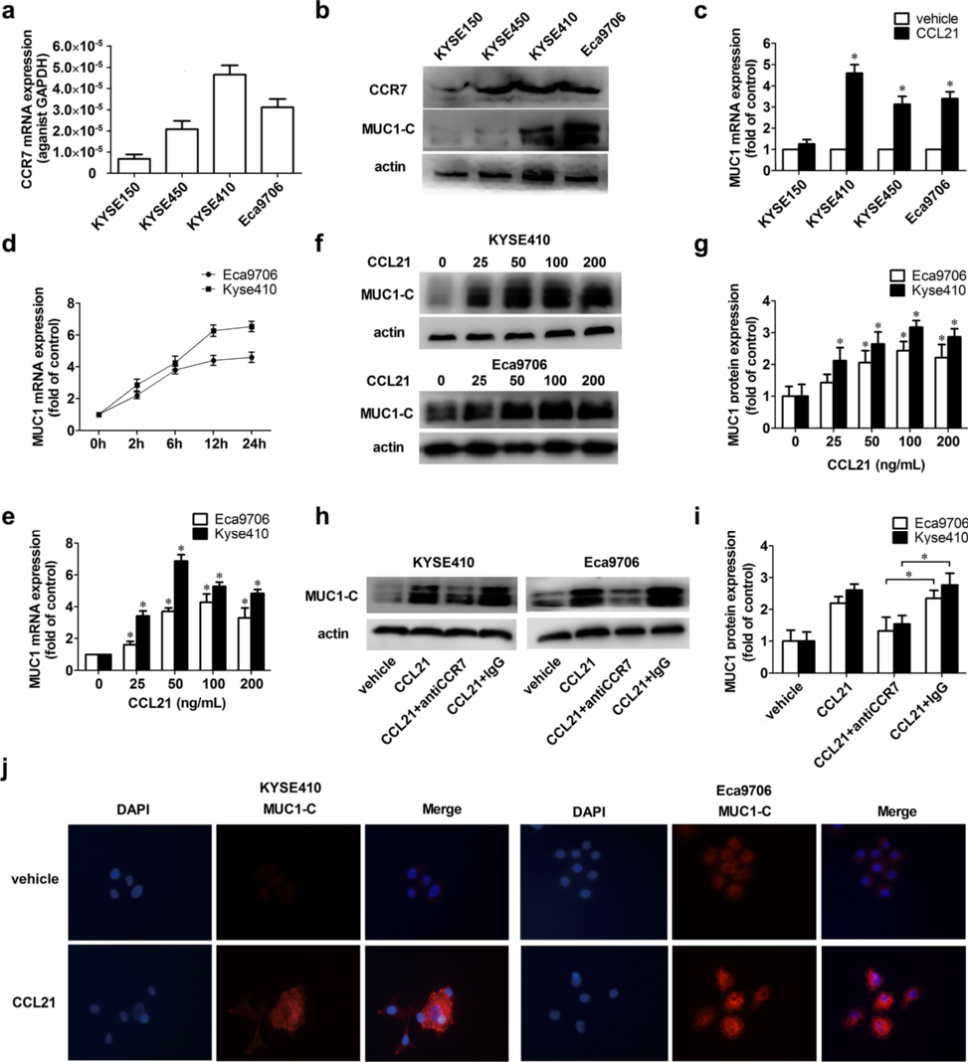
CCL21 induced the up-regulation of MUC1 in ESCC cell lines. **a**, **b** the mRNA and protein expression level of MUC1 and CCR7 in ESCC cell lines; **c**: CCL21 induced the increasing MUC1 mRNA in ESCC cells. KYSE150, KYSE410, KYSE450 and Eca9706 were starved for 24 h and then treated with CCL21 at a concentration of 100 ng/ml for 12 h, then cells were harvested for the qRT-PCR; **d**, **e** CCL21 up regulated mRNA of MUC1 in a time and dose dependent way. Eca9706 and KYSE410 were starved for 24 h and then treated with CCL21 at a concentration of 0, 25, 50, 100, 200 ng/ml for 12 h or cells were treated with CCL21 at a concentration of 100 ng/ml for 0, 2, 6, 12 or 24 h. Then cells were harvested for qRT-PCR; **f**, **g** increasing of MUC1-C in KYSE410 and Eca9706 after treated with CCL21 at concentration of 0, 25,50, 100, 200 ng/ml for 24 h confirmed by immunoblotting; **h**, **f** blocking CCR7suppressed the up-regulation of MUC1 induced by CCL21. Eca9706 and KYSE410 were pretreated by CCR7 antibody (1 ug/mL) or IgG as control, then treated by 100 ng/ml for 24 h, then cells were harvested for detection of MUC1 by immunoblotting; **j** expression of MUC1 in KYSE410 and Eca9706 treated with PBS or CCL21(100 ng/mL) detected by immunofluorescence. Each data point represents the mean ± SD of three repeated experiments. *P < 0.05

First, we detected the expression of MUC1 and CCR7 in KYSE150, KYSE410, KYSE450 and Eca9706 cells: higher mRNA and protein expression levels of CCR7 and MUC1 were found in KYSE410 and Eca9706, while the lowest expression levels of CCR7 and MUC1 were detected in KYSE150 (Fig. 2a, b). We then wondered whether activating CCR7 by its specific ligands could regulate the expression of MUC1. The ESCC cell lines were treated with CCL21 (100 ng/mL), and qRT-PCR showed a remarkable increases in the mRNA level in KYSE410, KYSE450 and Eca9706 cells with a higher expression level of CCR7, while there was no significant change of MUC1 in KYSE150 with a lower expression level of CCR7 (Fig. 2c). These results above were consistent to the co-expression of MUC1 and CCR7 in ESCC tissue samples. Furthermore, we found that CCL21 could increase the mRNA level of MUC1 in a dose- and time-dependent manner in KYSE410 and Eca9706 cells, and the saturated dose was approximately 50–100 ng/ml. The increase of MUC1 mRNA was detectable approximately 2 h after being treated with CCL21, and the highest level was detected at approximately 12–24 h (Fig. 2d, e). The western blot also confirmed that CCL21 could increase the expression of MUC1-C protein in a dose-dependent manner (Fig. 2f, g). To further confirm the role of CCR7 in the up-regulation of MUC1 induced by CCL21, we used the specific antibody to block CCR7, and the results showed that blocking CCR7 could significantly suppress the up-regulation of MUC1-C induced by CCL21 (Fig. 2h, i).

### CCL21-CCR7 promoted the migration and invasion of ESCC cell lines in vitro

**Fig. 3:**
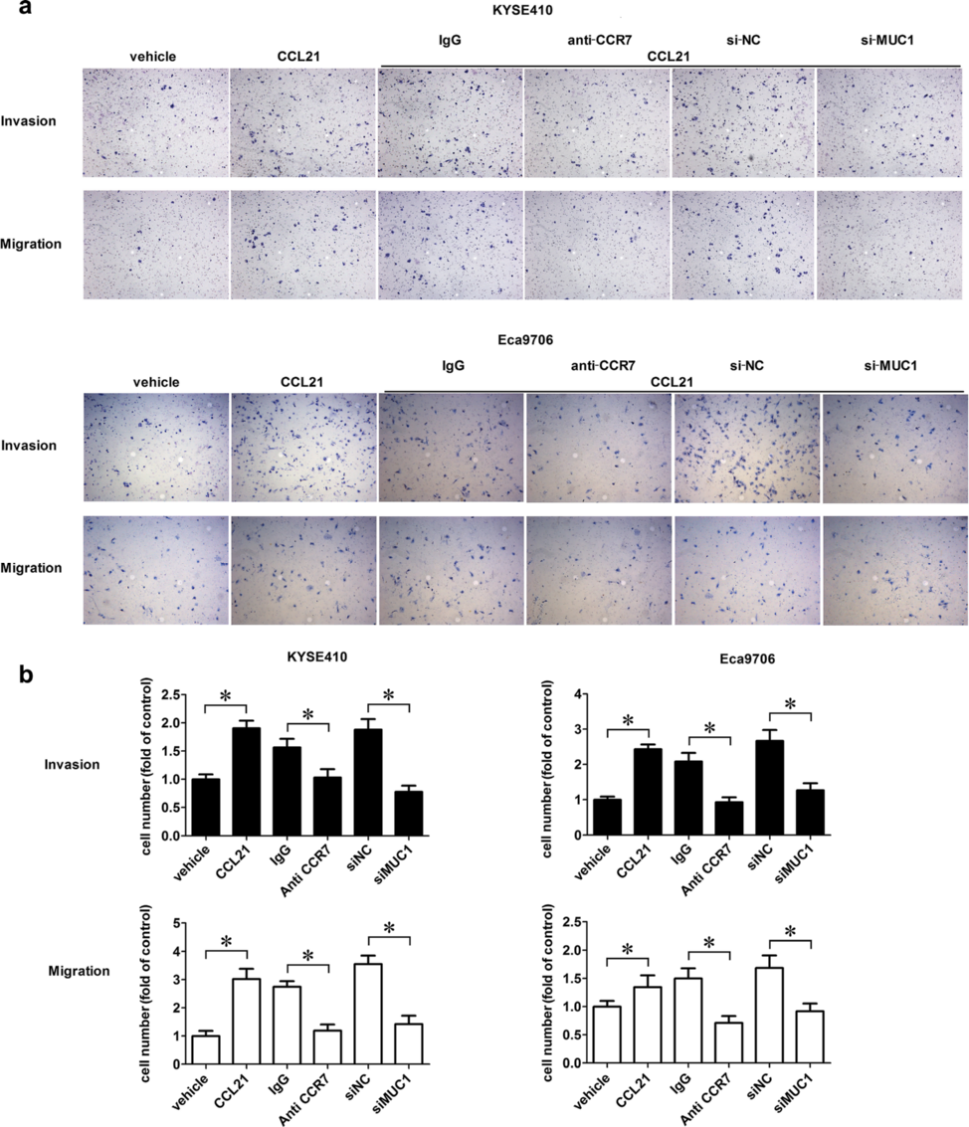
Silencing of MUC1 suppressed migration and invasion induced by CCL21. Cells were starved for 24 h and then seeding into the upper chamber, for the migration assay CCL21 was added into the lower chamber at a concentration of 200 ng/ml and incubated for 12 h; for the invasion assay the CCL21 was added into the upper chamber at a concentration of 200 ng/ml and incubated for 36 h. Cells on the lower surface of the membrane were counted in five randomly selected fields. **a** CCL21 promoted migration and invasion of KYSE410 and Eca9706 while blocking CCR7 could reverse migration and invasion induced by CCL21, and silencing MUC1 by siRNA targeted to MUC1 significantly suppressed the migration and invasion induced by CCL21; **b** Total cell numbers on the lower surface of the membrane counted in five randomly selected fields. Each data point represents the mean ± SD of 3 repeated experiments. *P < 0.05; Each datapoint represents the mean ± SD of three repeated experiments. *P < 0.05

Because the migration and invasion abilities of tumor cells are crucial for metastasis, we performed a transwell assay to evaluate the effect of the CCL21-CCR7 axis on the migration and invasion of KYSE410 and Eca9706. The results showed that CCL21 could significantly enhance the migration and invasion of KYSE410 and Eca9706. Furthermore, blocking CCR7 with the specific antibody (R&D, USA) significantly suppressed the migration and invasion of KYSE410 and Eca9706 induced by CCL21 (Fig. 3).

### Silencing MUC1 inhibited the migration and invasion of ESCC cell lines induced by CCL21

We have proven that the CCL21-CCR7 axis can promote the migration and invasion of ESCC cells *in vitro* and also increase the level of the MUC1-C protein. Considering MUC1-C as an oncogene in cancers, we assumed that the increasing expression of MUC1-C may be responsible for the migration and invasion induced by CCL21. Therefore, we established siMUC1-KYSE410 and Eca9706 for transwell assays and the results showed that silencing MUC1 could remarkably suppress the migration and invasion induced by CCL21 (Fig. 3).

### CCL21-CCR7 increased the up-regulation of MUC1 via activating ERK1/2

**Fig. 4:**
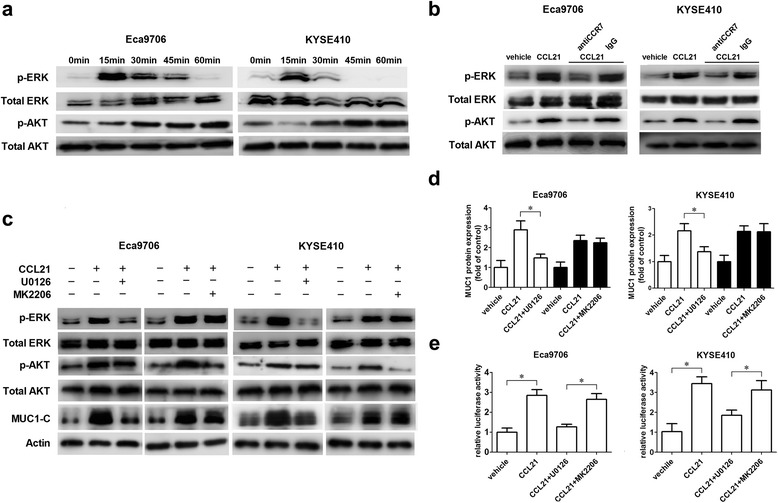
Activation of ERK1/2 was responsible for the up-regulation of MUC1 induced by CCL21-CCR7. **a** The activation of ERK1/2 and Akt pathway induced by CCL21, KYSE410 and Eca9706 cells were seeding into 6 well culture plate and starved overnight, then culture medium was replaced by serum free medium contained CCL21 (100 ng/ml) and incubated for 0, 15, 30, 45, 60 min. Then cells were harvested for immunoblotting; **b** Blocking CCR7 could suppress the activation of Akt and ERK1/2 pathway induced by CCL21 in KYSE410 and Eca9706; **c**, **d** Inhibiting activation of ERK1/2 but not Akt could suppress the up-regulation of MUC1-C protein. The starved KYSE410 and Eca9706 cells were pretreated by DMSO as controll, U0126 or MK2206 for 30 min, then cells were treated with PBS or CCL21 (100 ng/ml). For detecting p-ERK1/2, p-Akt and MUC1-C, cells were harvested after incubated with CCL21 for 15 min, 30 min and 24 h respectively; **e** Inhibiting ERK1/2 but not Akt could remarkably suppress the activity of MUC1 promoter, KYSE410 and Eca9706 cells transfected with MUC1-pGL2b plasmid were pretreated with DMSO as control, U0126 or MK2206 and then treated with PBS or CCL21 (100 ng/ml) for 12 h, then cells were harvested to detect the relative luciferase activity. Each datapoint represents the mean ± SD of three repeated experiments. *P < 0.05

We detected the increasing phosphorylation of Akt1 and ERK1/2 in KYSE410 and Eca9706 after treatment with CCL21, while blocking CCR7 could remarkably suppress the activation of ERK1/2 and Akt induced by CCL21 (Fig. 4a, b). To identify whether Akt1 and ERK1/2 pathway were responsible for the up-regulation of MUC1, KYSE410 and Eca9706 cells were pretreated with the specific inhibitor of Akt(MK2206) or ERK1/2(U1026), and the results showed that U1026 remarkably suppressed the up-regulation of MUC1 induced by CCL21, while MK2206 did not significantly suppress the up-regulation of MUC1 (Fig. 4c). Moreover, the luciferase reporter assay showed that inhibiting the activation of ERK1/2 but not Akt could suppress the activity of the MUC1 promoter after treatment with CCL21 (Fig. 4d). These results confirmed that the activation of ERK1/2 was responsible for the up-regulation of MUC1 induced by the CCL21-CCR7 axis.

### Activation of SP1 induced by p-ERK1/2 was responsible for the increasing transcription of MUC1 gene

**Fig. 5:**
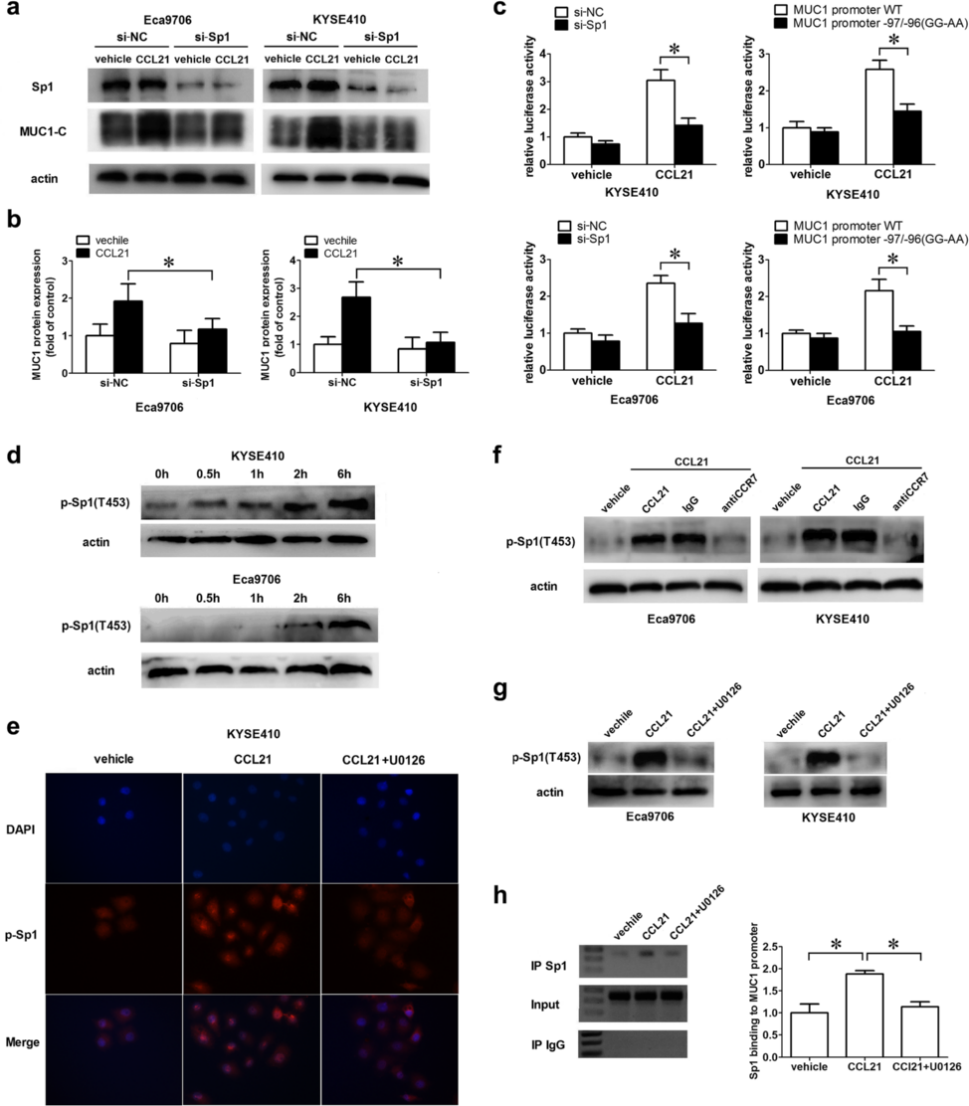
Phosphorylation of SP1 was responsible for the up-regulation of MUC1 induced by CCL21-CCR7. **a**, **b** Silencing Sp1 could remarkably suppressed the up-regulation of MUC1 in KYSE410 and Eca9706 induced by CCL21. The starved siSp1-Eca9706, siNC-Eca9706, siSp1-KYSE410 and siNC-KYSE410 were treated with CCL21(100 ng/mL) for 24 h, then cells were harvested for immunoblotting for MUC1-C; **c** Silencing Sp1/Mutant of Sp1 binding site at −99/−97 could remarkably suppressed MUC1 promoter activity induced by CCL21. siSp1-Eca9706, siNC-Eca9706, siSp1-KYSE410 and siNC-KYSE410 transfected by MUC1-pGL2b luciferase reporter plasmid/KYSE410 and Eca9706 cells transfected by MUC1-pGL2b or MUC1 mutant-pGL2b -firefly luciferase reporter plasmid were treated with PBS or CCL21(100 ng/mL) for 24 h, then cells were harvested for detecting the luciferase activity; **d** Increasing phosphorylation of Sp1 induced by CCL21. Starved KYSE410 and Eca9706 cells were treated with CCL21(100 ng/mL) for 0, 0.5, 1,2 and 6 h, then cells were harvested for the immunoblot of p-Sp1; **e** The expression of p-Sp1 in KYSE410 treated with PBS or CCL21(100 ng/ml) for 6 h detected by immunofluorescence; **f** Blocking CCR7 could suppress the phosphorylation of Sp1 induced by CCL21. The starved KYSE410 and Eca9706 cells pretreated with the CCR7 antibody or IgG as control, were treated with PBS or CCL21 for 6 h, then cells were harvested for the immunoblot of p-Sp1; **g** Inhibiting ERK1/2 suppressed phosphorylation of SP1 induced by CCL21. Starved KYSE410 and Eca9706 cells were pretreated with DMSO as control or U0126 for 30 min. After treated with PBS or CCL21(100 ng/mL) for 6 h, the cells were harvested for immunoblot; **h** Inhibiting ERK1/2 suppressed Sp1 binding to MUC1 promoter at −99/−97. Starved KYSE410 cells were pretreated with DMSO as control or U0126 for 0.5. After treated with PBS or CCL21(100 ng/mL) for 12 h, the cells were harvested for the ChIP assay. The targeted DNA was amplified using MUC1 primers with 40 cycles of PCR. Each datapoint represents the mean ± SD of three repeated experiments. *P < 0.05

Though our results showed that p-ERK1/2 was responsible for the up-regulation of MUC1 transcription, the underlying molecular mechanism still remained unclear. Several studies revealed that the regulation of MUC1 expression occurs mainly on the transcriptional level and that the SP1 binding site at −99/−90 on the promoter is crucial for the transcription of MUC1 gene [20, 21], and several fundamental works have proven that the phosphorylation of SP1 by Erk/Akt/PKC could regulate its DNA binding ability [22]. Based on this, we explored the role of Sp1 in the up-regulation of MUC1 induced by CCL21-CCR7. The results showed that silencing Sp1 could abolish the increase of MUC1 induced by CCL21 (Fig. 5a, b); consistently, the mutant of the Sp1 binding site on the MUC1 promoter at −97/−96(GG → AA) significantly suppressed the MUC1 promoter activity induced by CCL21 (Fig. 5c). These results confirmed the important role of Sp1 in the CCL21-CCR7-induced up-regulation of MUC1. Then, an increasing phosphorylation level of Sp1 at T453 (p-Sp1) was detected and the accumulation of p-Sp1 in nuclei after treatment with CCL21 was observed in KYSE410 (Fig. 5d, e), and inhibiting p-ERK1/2 could significantly suppress the phosphorylation of Sp1 and the accumulation of p-Sp1 in nuclei (Fig. 5f, g). Subsequently, the ChIP assay confirmed an increased level of SP1 binding to the MUC1 promoter in the CCL21-treated group compared to the control group, while inhibiting the activation of ERK1/2 could remarkably suppress the binding of SP1 to the MUC1 promoter at −99/−90 in KYSE410 (Fig. 5h). All of this evidence indicated that the ERK1/2-Sp1 pathway is involved in the up-regulation of MUC1 induced by CCL21-CCR7.

### Heterologous expression of CCR7 promoted the migration and invasion induced by CCL21 and also up-regulation of MUC1

**Fig. 6:**
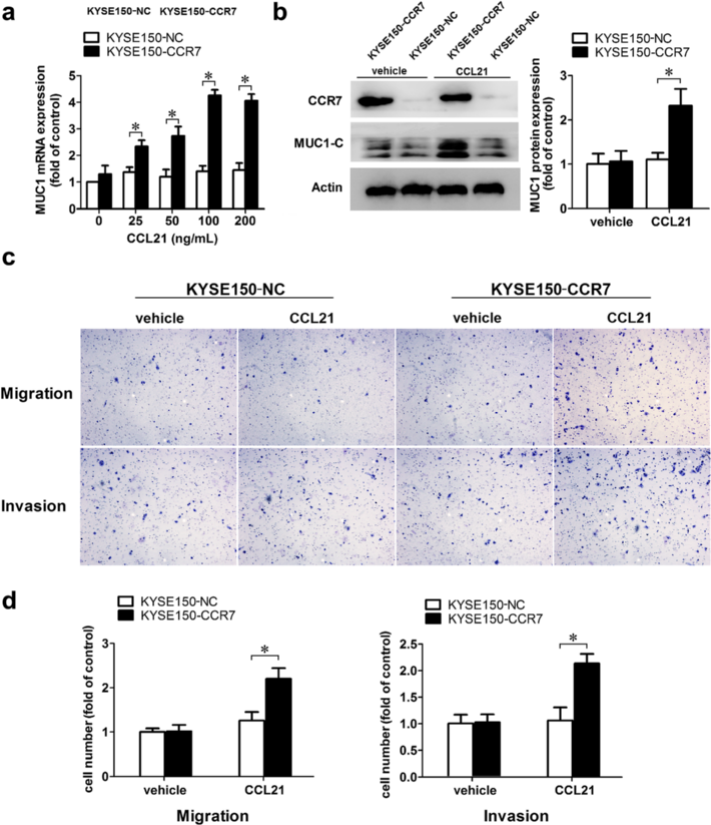
Heterologous CCR7 promoted migration and invasion and up-regulated expression of MUC1 in KYSE150. **a** Heterologous expression of CCR7 up regulated the expression of MUC1 mRNA in KYSE150, KYSE150-CCR7 and KYSE150NC cells were starved and then treated with PBS or CCL21(0, 25, 50, 100, 200 ng/mL) for 12 h and then harvested for qRT-PCR and the result showed the remarkable up-regulation of MUC1 in KYSE150-CCR7 after treated with CCL21 compared to the KYSE150NC groups; **b** Heterologous expression of CCR7 up regulated the expression of MUC1-C protein in KYSE150, KYSE150-CCR7 and KYSE150NC cells were starved and then treated with PBS or CCL21(100 ng/mL) for 24 h and then harvested for immunoblot and the result showed the remarkable up-regulation of MUC1 in KYSE150-CCR7 after treated with CCL21 compared to the KYSE150NC groups; **c** Heterologous expression of CCR7 promoted migration and invasion induced by CCL21, the starved KYSE150-CCR7 and KYSE150NC cells were seeding into the upper chamber, for the migration assay CCL21 was added into the lower chamber at a concentration of 200 ng/ml and incubated for 12 h; for the invasion assay the CCL21 was added into the upper chamber and incubated for 36 h; **d** Total cell numbers on the lower surface of the membrane counted in five randomly selected fields. Each data point represents the mean ± SD of three repeated experiments. *P < 0.05

In this study, KYSE150 had the lowest CCR7 and MUC1 expression levels among ESCC cell lines. To further explore the role of CCL21-CCR7 in promoting migration and invasion and its role in inducing the expression of MUC1, we established KYSE150-CCR7 and KYSE150-NC by transfecting lentivirus containing cDNA of the CCR7 gene or a nonsense sequence as a control. The transwell assay showed that CCL21 remarkably promoted the migration and invasion of KYSE150-CCR7 compared to the KYSE150-NC groups. Furthermore, a remarkable increase in the expression of MUC1 was also observed in the KYSE150-CCR7 compared to the KYSE150 NC groups after treatment with CCL21 by qRT-PCR and immunoblotting (Fig. 6).

## Discussion

As a highly aggressive malignant tumor, the major risk factor influencing the prognosis of ESCC patients is lymph node metastasis, and the most common reason for the failure of surgical treatment of ESCC is metastatic recurrence in regional lymph nodes. The metastasis of tumor cells is a complex process involving multiple key steps, and directional migration to the targeted organs is essential to achieve metastasis. The lymphatic metastasis of tumor cells is quite similar to the “homing” of mature lymphocytes to the secondary lymph node: with the guide of chemokine receptors, the lymphocytes or tumor cells could directionally migrate into targeted organs [5, 23]. As a chemokine receptor, CCR7 has been proven to be involved in lymphatic metastasis in various cancers, including ESCC [24, 25], and the role of the CCL21-CCR7 axis in inducing the directional migration and invasion of tumor cells has also been proven [26–29]. Consistent with these former studies, our results also showed that CCR7 was correlated with lymphatic metastasis, lymphatic recurrence status and poor prognosis in ESCC. And we also confirmed that CCL21 could promote the migration and invasion of the ESCC cell lines KYSE410 and Eca9706 *in vitro*. These findings confirmed the function of the CCL21-CCR7 axis in promoting lymph metastasis in ESCC.

The aberrant overexpression of MUC1 has been detected in ESCC and is correlated with lymphatic metastasis and poor prognosis [17, 30]; Qing Y et al. further confirmed that MUC1-C could promote lymph node metastasis in ESCC by up-regulating the expression of MMP13 [31]. Here we also confirmed that overexpression of MUC1 correlated with lymph node metastasis and recurrence in ESCC patients. Though MUC1 is a heterodimer protein localized on the cellular membrane, many fundamental works have confirmed the constant accumulation of MUC1-C in the cytoplasm, nuclei and mitochondria of cancer cells due to the MUC1-autocleavage [15] and further revealed its role in promoting metastasis by inducing epithelial mesenchymal transition (EMT), promoting cell motility, and up-regulating MMPs and uPA [32–35]. We also detected the localization of MUC1 in the nuclei and cytoplasm in the ESCC tissue samples and the cell lines, and this indicates the possible accumulation of MUC1-C in ESCC. However, the biological significance of the subcellular localization of MUC1 in ESCC still needs to be explored.

Though the CCL21-CCR7 axis may promote the aggressive biological behavior of ESCC cells, the key downstream molecules that regulate migration and invasion are still unclear. Here, we revealed the potential role of MUC1 in CCL21-CCR7-induced lymphatic metastasis in ESCC. By analyzing the expression of CCR7 and MUC1 in 153 ESCC tissue samples, we found the co-expression of CCR7 and MUC1 in ESCC, and the two molecules were both correlated with lymph node metastasis and prognosis; furthermore, the patients who co-expressed CCR7 and MUC1 had the highest recurrence rate and the lowest survival rate compared with the rest of the groups. Therefore, we speculated that there might be CCR7-MUC1 crosstalk in the progression of metastasis. Then, we identified the up-regulation of MUC1 mRNA and the MUC1-C protein in Eca9706 and KYSE410 after treatment with CCL21, and subsequent transwell assay confirmed that silencing MUC1 remarkably inhibited the migration and invasion of ESCC cell lines induced by CCL21 *in vitro*. Moreover, KYSE150 cells with the heterologous expression of CCR7 exhibited not only more aggressive biological behavior but also increasing MUC1 after being treated with CCL21. All of these results indicated that CCR7 could promote metastasis via the up-regulation of MUC1 in ESCC, and the molecular mechanism of MUC1 in promoting migration and invasion induced by CCL21- CCR7 in ESCC still requires further investigation.

Research has revealed that the Akt and ERK pathways are involved in the CCR7-induced migration of tumor cells [36–39], and we also detected increased activation of ERK1/2 and p-Akt in KYSE410 and Eca9706 cells after treatment with CCL21. To identify whether activated ERK1/2 and Akt were responsible for the up-regulation of MUC1 induced by CCL21, the ESCC cell lines was pretreated with the corresponding specific inhibitor. The results showed that inhibiting the activation of ERK1/2 could significantly suppress the transcription of the MUC1 gene, and Koga T et al. also reported the up-regulation of MUC1 induced by the TNFR-mediated activation of ERK1/2 in lung cancer cells [40]. Though Liao G et al. reported that the EGFR-mediated activation of the PI3k-Akt pathway could promote the transcription of the MUC1 gene [41], in this study, inhibiting the activation of Akt1 by MK2206 did not remarkably affect the expression of MUC1 *in vitro*.

Although our work proved that activated ERK1/2 was responsible for the increased transcription of MUC1 induced by CCL21-CCR7, the downstream signaling pathway is still unclear. Previous studies have revealed that the binding of SP1 to the MUC1 promoter at −99/−90 is crucial for the transcriptional regulation of MUC1 gene, and the phosphorylation of SP1 by ERK1/2 could alter its binding affinity to the promoter of certain genes, such as VEGF and erbB2, for transcriptional regulation [42, 43]. Therefore, we assumed that the ERK1/2-Sp1 pathway is involved in the up-regulation of MUC1 gene transcription induced by CCL21-CCR7. Correspondingly, we found increased phosphorylation of SP1 at T453 and subsequent increases in Sp1 binding to the MUC1 promoter at −99/−90 after treatment with CCL21 in KYSE410. And inhibiting the activation of ERK1/2 remarkably suppressed the phosphorylation of SP1 at T453 and the binding of SP1 to the MUC1 promoter. All of these evidences indicated the involvement of the ERK1/2- SP1 pathway in the CCL21-induced expression of MUC1.

## Conclusion

In conclusion, our study confirmed the role of MUC1 in lymph node metastasis induced by CCL21-CCR7 in ESCC and elucidated the possible phosphorylation of the ERK1/2 and Sp1 pathways in the regulation of MUC1 by CCL21-CCR7. Our results may help elucidate the molecular mechanism of lymph node metastasis in ESCC and offer a theoretical basis for targeted therapy.

### Ethics, consent and permissions

This study was carried out in strict accordance with the recommendations in the Guide for the Chinese Ethics Review Committees. The protocol was approved by the Ethics Committee of Provincial Hospital Affiliated to Shandong University.

## Abbreviations

CCR7: C-C motif chemokine receptor type 7
CCL19/21: C-C motif chemokine ligand19/21
ChIP: chromatin immunoprecipitation
EMT: epithelial mesenchymal transition
ESCC: esophageal squamous cell carcinoma
IHC: immunohistochemistry
IHS: immunohistochemical score
MMPs: matrix metalloproteinases
MUC1: cell surface associated mucin1
RT-PCR: reverse transcription polymerase chain reaction
SD: standard deviation
